# Novel Surgical Initiatives in Gastroenteropancreatic Neuroendocrine Tumours

**DOI:** 10.1007/s11912-024-01632-4

**Published:** 2025-01-25

**Authors:** Alina S. Ritter, Jelte Poppinga, Kira C. Steinkraus, Thilo Hackert, Anna Nießen

**Affiliations:** Department of General, Visceral and Thoracic Surgery, University Medical Center Hamburg- Eppendorf, Martinistraße 52, D-20246 Hamburg, Germany

**Keywords:** Neuroendocrine tumour, Pancreatic neuroendocrine tumour, Intestinal neuroendocrine tumour, GEP-NET, Oncologic surgery

## Abstract

**Purpose of Review:**

Neuroendocrine tumours (NET) are rare entities arising from hormone producing cells in the gastroentero-pancreatic (GEP) tract. Surgery is the most common treatment of GEP-NETs.

**Recent Findings:**

Improvements in surgical techniques allow for more locally advanced and metastasised GEP-NETs to be resected. Laparoscopic and robotically--assisted approaches are increasingly being utilised in the resection of selected GEP-NETs and are facilitated by novel intraoperative tumour localisation tools and parenchyma-sparing methods. At the same time, some authors suggest that indications for formal resections of small well differentiated non-functioning pancreatic NETs and appendiceal NETs should be more restrictive.

**Summary:**

Advancements in surgery allows for tissue-sparing resections of GEP-NETs. Indications for surgical resection and the extent of the procedure are highly dependent on GEP-NET size, localisation and grading. Robotically assisted surgeries with intraoperative ultrasound and visualisation methods as well as vessel-sparing radical retrograde lymphadenectomies for small intestinal NETs seem to be the future of GEP-NET surgery.

## Introduction

Neuroendocrine tumours (NETs) are rare neoplasms, deriving form hormone-producing cells, with a rising incidence [1]. The most frequent localisation of NETs is the lung, followed by the gastroentero-pancreatic system. Surgical resection remains the most common treatment for gastroentero-pancreatic NETs (GEP-NET) [2]. Current guidelines by the European Neuroendocrine Tumor Society (ENETS) and the North American Neuroendocrine Tumor Society (NANETS) recommend resection depending on tumour localization, tumour size, proliferation index and functional activity [3, 4, 5, 6, 7, 8, 9, 10]. Apart from surgery, somatostatin analogues (SSAs), peptide receptor radionuclide therapy (PRRT) and chemotherapy are the main pillars in modern GEP-NET treatment [3, 4, 5, 6, 7, 8, 9, 10]. In case of non-resectable metastasis local ablative treatments such as microwave ablation (MWA) or radiofrequency ablation (RFA) as well as transarterial approaches like transarterial (chemo-)embolization (TA(C)E) and selective internal radiotherapy (SIRT) can be applied [5, 7].

Prognosis of localised, well-differentiated GEP-NETs is excellent after R0 resection [5, 6, 7]. Simultaneously, the survival benefits of a R0 resection need to outweigh the risks and complication rates of surgery [11]. Severe complications occur in approximately 14.9% after GEP-NET resections, but are highly dependent on the anatomic region, with pancreatic surgery being associated with the highest complication rates [11]. Selection of the appropriate surgical procedure and resection technique based on preoperative imaging and the current literature for the individual patient remains the key challenge in surgery for GEP-NETs.

This review aims to give an overview over the recent advancements in GEP-NET surgery focusing on current indications for resection, the appropriate resection extent and especially novel technological developments in order to improve GEP-NET resections.

## Pancreatic NETs – to Resect or Not to Resect that is the Question

### Surveillance of Pancreatic NETs ≤ 2 cm

The incidence of pancreatic NETs (pNETs) is rising and with improvements of imaging modalities, even smaller lesions can nowadays be identified [1]. The survival benefit of surgery needs to be weighed against the morbidity and potential mortality that is associated with pancreatic surgery. European and North-American Guidelines have recommended surgical resection of non-functioning pNETs (nf-pNETs) only from a size cutoff ≥ 2 cm, however, these recommendations are based on retrospective study designs and remain debated [4, 12, 13]. To improve the strength of evidence for the optimal treatment of small, nf-pNETs, a Dutch multi-centre (PANDORA) and an international (ASPEN), prospective observational trial have been initiated [14, 15]. The interim analyses of both studies published thus far, found a watch-and-wait strategy with regular long-term follow-up to be feasible and safe despite a risk of tumour progression with need for surgery between 2% and 11% respectively for pNETs ≤ 2 cm [14, 15]. Thus, the final results of these studies need to be awaited before definitive conclusions can be drawn.

Indication for resection of small nf-pNETs may be restricted to high Ki-67 (higher grading), tumour growth, pathological lymph node enlargement, vascular involvement, signs of local infiltration, pancreatic duct dilatation, distant metastasis or patient request. In contrast, a retrospective analysis of the American National Cancer Database found a significantly improved 5-year overall survival in patients, who underwent resection of pNETs between 1 and 2 cm. This benefit was, however, not detectable after resection of even smaller pNETs (< 1 cm) [16]. Additionally, enucleation of small nf-pNETs < 3 cm is a safe and feasible procedure with similar complication rates and comparable long-term results [17].

Due to these controversial findings, current ENETS guidelines recommend active surveillance for nf-pNETs ≤ 1 cm and a personalized approach for nf-pNETs between 1 and 2 cm in size, whereas main pancreatic duct dilation and a tumour size > 2 cm are generally indications for surgical resection [5].

### Parenchyma Sparing vs. Formal Resection of Pancreatic NETs

Parenchyma-sparing as well as formal oncological resections are both well-established surgical concepts in treating pNETs. The risk of metastases and recurrence has to be balanced with the risk of overtreatment, potential postoperative complications as well as exocrine and endocrine pancreatic insufficiency due to more radical resections. Thus, there is an ongoing debate on the adequate extent of pNET resections and the role of systematic lymphadenectomy also for localised approaches.

In functionally-active pNETs (f-pNETs), indications for the respective approaches are well defined: Insulinomas, the most common type of f-pNET, can safely be resected with parenchyma-sparing procedures such as enucleation without routine lymphadenectomy, since the risk of malignancy is low [6, 18, 19]. In contrast, gastrinomas require formal resection with adequate lymphadenectomy, as lymph node metastases are present in 80% of patients, even in very small (< 5 mm) gastrinomas and tumours thus have a more malignant behaviour [4, 6].

The appropriate approach for nf-pNETs is, however, less well-defined. Whilst formal resection with systematic lymphadenectomy presents the gold standard for most malignant entities, the decision for formal resection and/or systematic lymphadenectomy in nf-pNET patients is based on tumour size, grading and the presence of metastases [5]. Additional risk factors for recurrence of nf-pNETs include perineural invasion, Chromogranin A levels, molecular factors and symptoms due to local tumour progression (e.g. jaundice, pain or bleeding) [5]. In small, well-differentiated nf-pNETs that are distant to the main pancreatic duct, enucleation can safely be performed with shorter operating times, lower rates of postoperative diabetes and non-inferior oncologic outcome [17, 20]. In some studies enucleation was associated with higher rates of postoperative pancreatic fistulas, however, not resulting in a higher postoperative overall morbidity and mortality [21, 22]. Simultaneously, the prognostic impact of lymph node metastases in nf-pNETs remains controversial: While some studies report an association between lymph node metastases and overall- and disease-free survival in pNETs, other studies challenge this concept or limit the prognostic impact to certain subgroups only [13, 23, 24, 25, 26, 27].

Currently, according to ENETS guidelines enucleation for nf-pNETs between 2 and 3 cm may be carefully considered depending on the patient’s individual risk constellation [5]. Furthers studies are needed to identify which patients with nf-pNET profit from formal resection, lymphadenectomy or lymph node picking only in contrast to low risk patients, when parenchyma-sparing resections are sufficient.

### Robotic Surgery for Pancreatic NETs

Over the last 20 years, robotic pancreatic surgery has advanced from a novel more or less experimental technique to a standard approach in high-volume pancreatic centres [28, 29]. A lot of experience has been gained in the robotic resection of the far more common pancreatic ductal adenocarcinoma (PDAC) and these technical advancements can likewise be applied in pNETs [30, 31]. In terms of oncological radicality, robotic resections are not inferior to laparoscopic or open approaches for PDAC [30, 31, 32]. Spleen-preserving procedures can more frequently be achieved in robotic distal pancreatectomies [33]. Simultaneously, minimally invasive resection of nf-pNETs is associated with a shorter hospital stay, a reduced complication rate and similar overall survival compared to open pancreatic resection [34, 35].

**Fig. 1:**
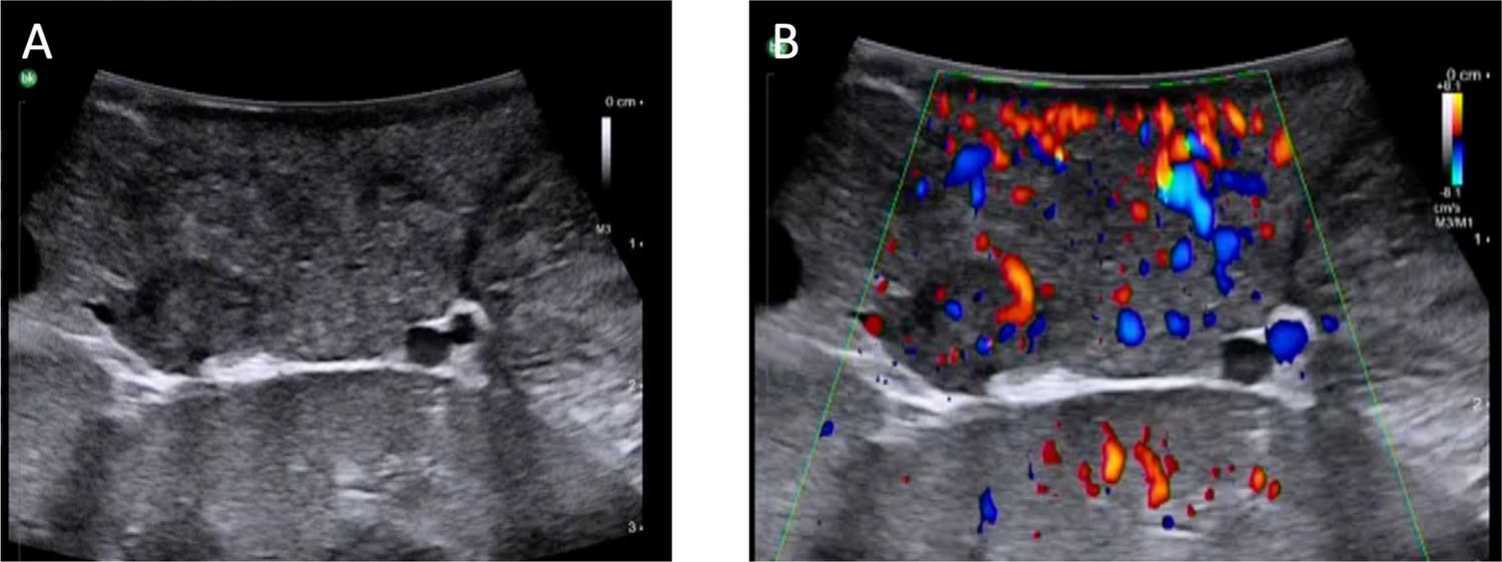
Intraoperative ultrasound (**A**) and colour-coded duplex sonography (**B**) during robotic resection of a well-differentiated insulinoma (arrows) in the pancreatic tail

A disadvantage of robotic surgery is the lack of haptic feedback making intraoperative tumour localisation by palpation not conductible, thus requiring precise preoperative imaging. Intraoperative ultrasound and tumour visualization tools are promising strategies to compensate for this drawback, if an NET cannot be visualized in preoperative cross-sectional imaging [36, 37] (Fig. 1). Novel approaches to facilitate intraoperative GEP-NETs localisation are outlined in detail below.

A recent meta-analysis compared minimally invasive with open resection of nf-pNETs and found a significantly shorter hospital stay and lower blood loss associated with minimally invasive surgery (MIS) [38]. Complication rates did not significantly differ, however there was a trend towards lower complication rates after MIS distal pancreatectomy [38]. Thus, MIS appears a safe and feasible approach for the resection of nf-pNETs. However, data on long-term results and oncologic outcomes, as well as randomised data is lacking.

Pancreatic NETs appear to be suitable indications for robotically-assisted procedures due to their – in comparison with PDAC – less aggressive growth patterns, however, specific data on robotic resection of pNETs is scarce. In experienced centres, robotic and laparoscopic resection of pNETs may be encouraged in suitable patients [5, 6].

### Portal Vein Resection of Locally Advanced Pancreatic NETs

Pancreatic tumours are non-functional in 50–85% of cases, frequently delaying initial diagnosis to an advanced tumour stage [5]. Surgical resection remains the treatment of choice for larger nf- and any size of functional-pNET [5]. As for PDAC in the pancreatic head local, resectability mostly depends on the degree of vessel involvement. The International Study Group of Pancreatic Surgery (ISGPS) has defined PDACs as resectable, borderline resectable, locally advanced and irresectable based on the involvement of the superior mesenteric vein (SMV) or portal vein (PV) as well as the celiac trunk (CT), hepatic artery (HA) and superior mesenteric artery (SMA) [39]. This classification has been transferred in clinical practice to describe the resectability of pNETs in the pancreatic head [5]. The advancements in pancreatic surgery in the last decades have resulted in an increased number of patients being eligible for tumour resection and higher rates of R0 resection [40]. Portal vein resection and reconstruction (PVR) has become a standard procedure in pancreatic centres [41, 42].

Major vessel involvement occurs in up to 17% of pNETs [43]. Based on the advancements in PDAC resection, PVR has been applied to locally advanced pNETs alike, however, data on oncologic benefit of these procedure remain scarce. Involvement of the SMV in locally advanced pNETs is not considered irresectable per se, if vascular resection is technically manageable and if no vein occlusion is present [12].

**Fig. 2:**
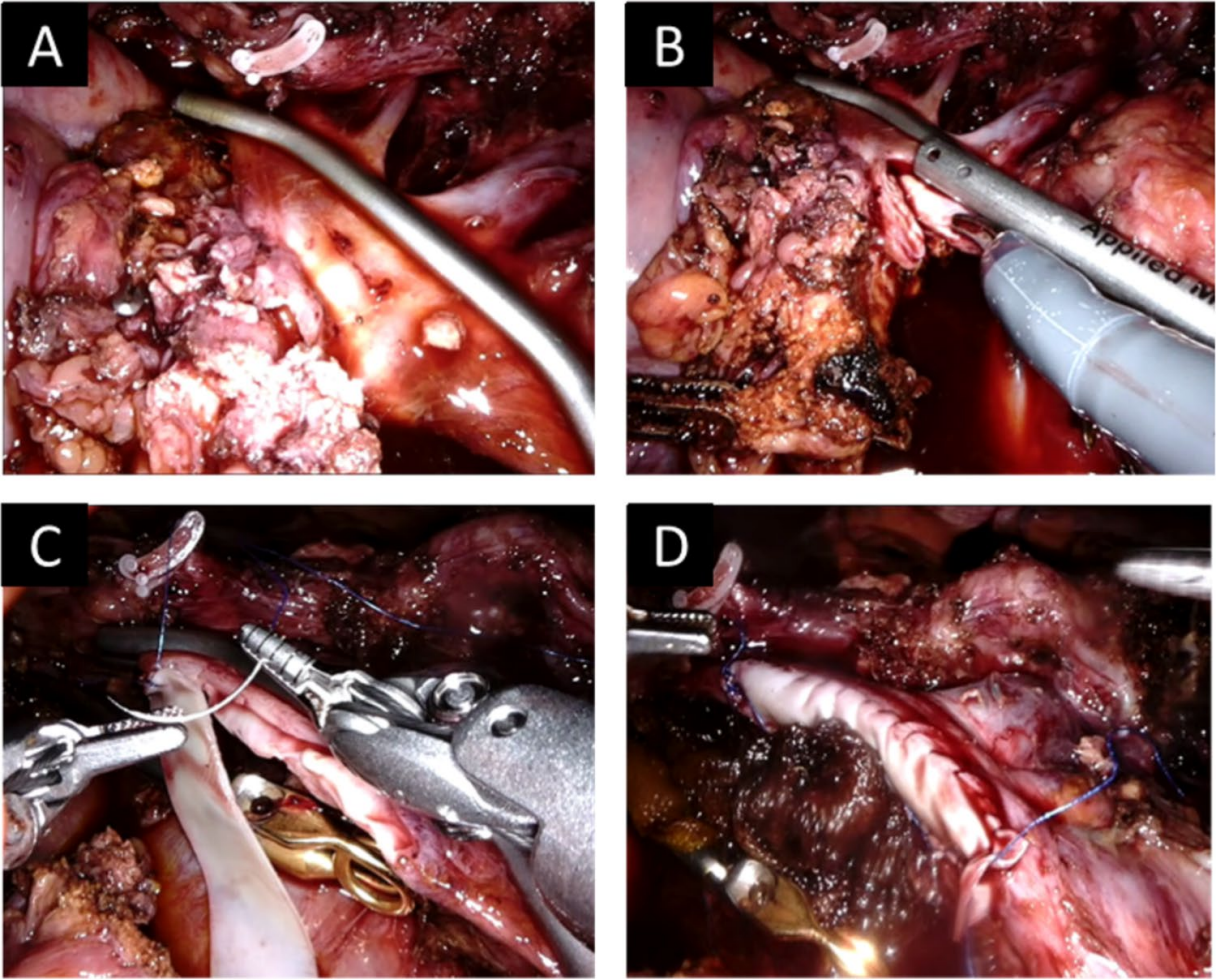
Intraoperative image of robotic portal vein resection (**A** and **B**) and Type II reconstruction (**C** and **D**) using a bovine pericardial patch. The figure is adapted from Kulu , Y. , Contin , P. , Hackert , T. (2021). Pankreaschirurgie. In: Hackert , T. , Croner , R.S. (eds) Roboterassistierte Viszeral- und Thoraxchirurgie. Springer , Berlin , Heidelberg. Figure 4.10; p. 41 [German]

Recently, three larger studies on pancreatic resection with PVR have been published [44, 45, 46]: Nießen et al., Fusai et al. and Gudmundsdottir et al. found no significant difference in postoperative complication rates as well as comparable overall- and disease-free survival after PVR compared to standard resection for pNETs after propensity-score matching. As expected, all observed higher blood loss and longer operation times when PVR was conducted [44, 45, 46]. Importantly, PVR is associated with the risk of developing portal vein thrombosis, especially when venous allografts are necessary (ISGPS Type IV reconstruction) [39, 41]. This type of reconstruction should thus be avoided whenever feasible. Close monitoring of postoperative liver perfusion is mandatory after these procedures, especially when an allograft was necessary [41]. There is no data on the recommended mode and duration of anticoagulation after PVR and each centre follows its own protocol [44]. In selected cases, PVR may even be performed robotically (Fig. 2).

Taken together, resection and PVR of locally advanced pNETs with portal vein involvement may be considered in selected patients. However, the conduction of such advanced procedures should be limited to experienced centres and only be conducted after interdisciplinary and thorough discussion with the individual patient [5].

### Primary Tumour Resection in Case of Distant Metastasis

For localised and locally advanced pNETs, primary tumour resection – with resection of potential metastasis – is the treatment of choice [4, 5, 6]. However, the benefit of primary tumour resection in case of distant metastasis remains ambiguous. Previously, only small studies evaluating the benefit of primary tumour resection were available [47, 48]. Recently, two larger studies retrospectively evaluated the benefit of primary tumour resection for stage IV pNETs. They could both show a benefit in overall survival for patients undergoing primary tumour resections [12, 49, 50].

Thus, primary tumour resection should be considered for stage IV pNETs, who are fit enough for surgery to reduce tumour burden and thereby potentially improve survival. However, prospective data on resection of stage IV pNETs is lacking [51]. Also, the potential benefit of neoadjuvant treatment, i.e. with PRRT, in order to enable a secondary resecability of advanced pNETs remains unclear [51, 52, 53].

**Fig. 3:**
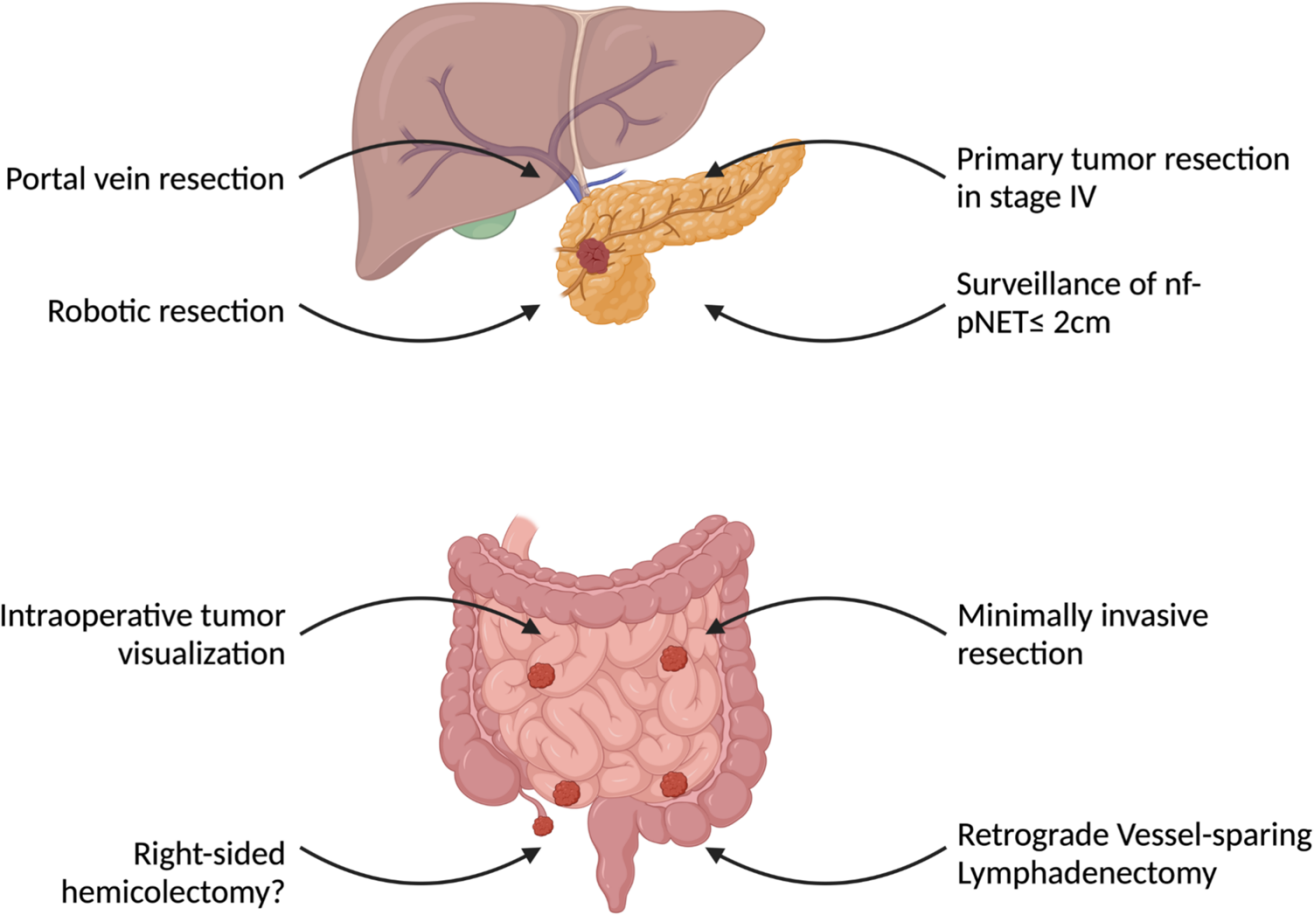
Schematic display of current indications for surgical resection of pancreatic, small intestinal and appendiceal neuroendocrine tumours. nf-pNET: non-functioning pancreatic neuroendocrine tumour. The figure was created in BioRender. Ritter , A. (2024) https://BioRender.com/z45h674

The novel surgical approaches of pNETs are summarized in Fig. 3.

## Small Intestinal NETs – How Should We Resect?

### Retrograde Vessel-Sparing Lymphadenectomy for Small-Intestinal NETs

The small intestine in the most frequent localisation of NETs in the gastrointestinal tract [1]. While usually small and slowly proliferating, small intestinal NETs (siNETs) are frequently locally advanced or metastasised to regional lymph nodes or the liver at the time of primary diagnosis [54, 55, 56]. Mesenteric lymph node metastasis can exceed the primary tumour in size and cause desmoplastic fibrosis, resulting in shrinkage of the mesentery [57]. This can cause abdominal pain, initiating a diagnostic work up in the first place or result in acute bowel obstruction, mesenteric ischemia or bleeding requiring emergency surgery [57, 58].

The standard of care for siNETs located > 20 cm orally of the ileocolic valve is the resection of the primary tumour as well as a systematic lymphadenectomy along the mesenteric vessels. This used to result in the resection of a “pizza pie”-shaped bowel segment with the adjacent mesenteric soft tissue [3, 7, 12, 59, 60]. A minimum of eight lymph nodes should be harvested to improve oncologic outcome. Recently, a newer study even suggested a minimum of 12 lymph nodes [12, 61, 62, 63]. However, due to the possibility of multifocal primary tumour localisation, local advancement of the primary tumour or central mesenteric lymph node metastases and their proximity to the intestinal blood vessels, extensive small bowel resection can be necessary to achieve a R0 resection. This puts the patient at risk of developing short bowel syndrome, resulting in malnutrition and diarrhoea and necessitating parenteral nutrition [55].

However, recent studies have shown that the length of the resected small bowel segment does not correlate with the number of lymph nodes harvested and lymph node metastases can skip in between lymph node levels [60, 64]. This has resulted in the proposal of “retrograde vessel-sparing lymphadenectomy” (VS-LA) in order to reduce the length of resected intestine, without sacrificing oncologic radicality [65, 66]. Technically, this is achieved by longitudinally incising the peritoneum along the SMA below the horizontal duodenum or pancreatic body and removing the adjacent soft tissue. Subsequently, retrograde dissection of the lymphatic tissue around the main mesenteric artery and vein distally of the tumour, while preserving ileocolic vessels, vascular collaterals and arcades along the intestine. Finally, the tumour affected bowel segment is resected as a last step [65].

A retrospective study comparing 25 patients with conventional vs. 25 with VS-LA showed, that shorter small bowel segments were resected in the VS-LA group, (40 (11-65) vs. 65 (23-190) cm), while the number of harvested lymph nodes as well as R0 resections were comparable. Severe postoperative complications (Clavien-Dindo ≥ 3), postoperative abdominal pain was less frequent in the VS-LA group. No local recurrence was observed in either group, however, long-term follow up was significantly shorter in the VS-LA group (24 (3–91) vs. 63 (6–94) months) [65]. Thus far, no larger studies are available to further evaluate the potential benefit of this resection technique [7].

### Right-Sided Hemicolectomy for NETs in the Distal Ileum

For NETs located proximally to the ileocolic valve, it remains controversial, when segmental bowel resection or formal right-sided hemicolectomy should be performed [3, 12, 59]. Interestingly, a study by Li et al. compared 93 patients receiving right hemicolectomy with 34 patients undergoing ileocecal resection for NETs in the distal ileum. While the number of harvested lymph nodes was significantly lower after ileocecal resection (17 vs. 14) - yet above the recommended minimum number of 8 – overall- (OS) and disease-free (DFS) survival did not significantly differ [67]. In contrast, Evers et al. found a right-sided hemicolectomy to be significantly associated with higher DFS in siNET patients [68].

Thus, even the most current ENETS guidelines for siNETs within 20 cm orally to the ileocolic valve still recommend either procedure depending on individual risk factors [7].

### Minimally Invasive Surgery for Small Intestinal NETs

The invention and further expansion of MIS has probably been the biggest paradigm-shifting development in surgical practice in the last 50 years. It is associated with reduced postoperative pain, shorter hospital stay and faster return to everyday life [69]. While MIS has gained wide acceptance for many procedures, laparoscopic resection of siNETs remains controversial and ENETS and NANTES guidelines recommend laparoscopic resection of siNETs in selected cases only [3, 7]. Disadvantages of MIS in the context of siNETs are the inability to perform bimanual palpation of the entire intestine – thereby missing potential multifocal tumour growth –, confined exposure of the abdominal cavity – thereby overseeing occult peritoneal lesions – and the restricted vascular control when performing central mesenteric lymphadenectomy – thereby performing less radical lymphadenectomy. Additionally, the presence of mesenteric fibrosis as well as acute intestinal obstruction, bleeding or ischemia may deem the patient unsuitable for laparoscopic resection [7].

Despite these potential obstacles, laparoscopic resection of siNETs has been increasingly conducted in high-volume centres: The lymph node yield may be either equal or even higher in the laparoscopically resected siNETs [70, 71, 72]. However, reported conversion rates range from 17.7 to 30% [70, 72]. As for other procedures, minimally invasive resection of siNETs was associated with a shorter hospital stay and a reduced rate of unplanned readmissions [70, 72]. Importantly, long term oncological outcomes reported so far, have been comparable between laparoscopic and open resection of siNETs [73].

Thus, MIS or hand-assisted MIS for siNETs is increasingly performed in experienced centres in elective settings taking into account the risk of intraoperative conversion especially in advanced tumour stages.

### Intraoperative Tumour Localization of Small Intestinal NETs

One of the unsolved problems in the diagnostics of siNETs and thus also the possibility of MIS, is the difficulty to achieve preoperative tumour localisation: Only siNETs in the very distal ileum can be visualized by colonoscopy. Additionally, primary tumour size is often below the resolution threshold of CT and MRI scans, which is further distorted by motion artefacts due to small intestinal peristalsis. Simultaneously, approximately 40–60% of siNETs grow multifocally and even primary tumours < 1 cm can cause lymph node metastasis [7]. Thus, bimanual palpation of the entire intestine remains the recommended diagnostic strategy [3, 7].

An approach to overcome these diagnostic challenges are intraoperative tumour visualization tools: Indocyanine green (ICG) is an easy to use, safe and widely available dye to perform intraoperative visualization; it illuminates at the excitation wavelength (about 750 to 800 nm) while emission wavelengths (over 800 nm) can be observed [74]. ICG can be used to visualize bowel perfusion intraoperatively. In the context of siNETs, this can either enable bowel sparing-resections as well as to prevent anastomotic reconstruction within a malperfused bowel segment [75]. Additionally, ICG can visualize superficial liver metastasis as well as facilitate R0 resections in parenchyma-sparing liver resections [76, 77]. Likewise, ICG has successfully been used in the localisation of pNETs [37].

An alternative approach to intraoperative tumour localization is near-infrared fluorescence autofluorescence (NIRAF). NIRAF was first discovered in parathyroid glands, which emit an autofluorescence signal when excited with a 785 nm wavelength laser [78]. A small feasibility study has applied NIRAF in siNET patients: The autofluorescence intensity of siNETs was significantly higher than the one of healthy small bowel and it was superior to DOTATATE-PET/CT and bimanual palpation in the detection of siNET primary tumours [76]. However, the benefit of NIRAF for detection of lymph node and peritoneal metastases was limited.

Radio-guided surgery is another approach using radioactive probes to localize tumours intraoperatively: In the context of siNETs, the expression of somatostatin receptors can be exploited using 68Ga-DOTATATE labelled probes to facilitate the localisation of primary siNETs as well as lymph node metastasis [79, 80]. Due to the obvious logistical efforts using radioactive tracer substances, this procedure has not gained widespread acceptance so far.

All mentioned approaches to improve intraoperative tumour localisation of GEP-NETs have been evaluated, however, currently experiences are still limited to small feasibility studies. The use of these detection tools still need validation in larger cohorts before any of these become widely applied in clinical practice.

The recent surgical advancements in siNETs are displayed in Fig. 3.

## Appendiceal NETs – Are We Overtreating?

### Omitting of Right-Sided Hemicolectomy for Appendiceal-NETs < 2 cm?

Appendiceal neuroendocrine tumours (aNETs) are found incidentally after about 1.5% of all appendectomies with a yearly incidence of 0.15–0.6 per 100.000 [81, 82]. Current ENETS guidelines recommend simple appendectomy for aNETs < 1 cm and oncological right-sided hemicolectomy for tumours > 2 cm [8, 82]. However, there is still a controversy regarding the adequate extent of operation of aNETs 1–2 cm in size. Currently, positive or unclear resection margins, deep mesoappendiceal invasion > 3 mm, high proliferation rate (G2), vascular (V1) or lymphatic vessel invasion (L1) are considered risk factors for lymph node metastasis and would thus necessitate a right hemicolectomy with lymph node dissection [82, 83]. However, recent studies have challenged this recommendation, resulting in potential overtreatment of the intermediate risk group [84, 85, 86, 87, 88].

A recent European retrospective, multi-centre study has compared 278 patients with aNETs between 1 and 2 cm, 163 of which were treated with an appendectomy and 115 with oncological right-sided hemicolectomy [89]. No differences were found in long-term survival between the two groups, suggesting that right-sided hemicolectomy may be omitted for aNETs ≤ 2 cm [89, 90]. No patient developed metachronous, distant metastasis of aNET in either group [89]. Thus, the indication for right-sided hemicolectomy might be re-evaluated in future NET guidelines.

## Conclusions

Surgery remains the most common treatment and only potential cure for GEP-NETs, however, when and how to operate on GEP-NETs has undergone substantial shifts in the last years. While for some entities a watch-and-wait approach or less radical surgery may be justified, evolution in surgical techniques also allow for locally advanced NETs to be resected in selected patients. Simultaneously, novel techniques such as minimal invasive and robot-assisted surgery, intraoperative imaging tools and vessel-sparing resections, reduce collateral tissue damage while maintaining appropriate oncologic radicality.

Surgical guidelines and techniques for resections of GEP-NETs need to be continuously challenged and revisited in order to continuously provide optimal interdisciplinary care for GEP-NET patients at specialised centres.

### Key References

- Partelli S, Massironi S, Zerbi A, Niccoli P, Kwon W, Landoni L, et al. Management of asymptomatic sporadic non-functioning pancreatic neuroendocrine neoplasms no larger than 2 cm: interim analysis of prospective ASPEN trial. Br J Surg. 2022;109 (12):1186-90.
  - This is an interim analysis of the largest multicentre, non-randomised, prospective trial that determines the outcome of resection versus active surveillance in small nf-pNETs.
- Heidsma CM, Engelsman AF, van Dieren S, Stommel MWJ, de Hingh I, Vriens M, et al. Watchful waiting for small non-functional pancreatic neuroendocrine tumours: nationwide prospective cohort study (PANDORA). British Journal of Surgery. 2021;108 (8):888 − 91.
  - This is an interim analysis of a large, prospective cohort study in the Netherlands evaluating the safety of a watch-and-wait approach in patients with nf-pNETs ≤ 2 cm.
- Klotz R, Mihaljevic AL, Kulu Y, Sander A, Klose C, Behnisch R, et al. Robotic versus open partial pancreatoduodenectomy (EUROPA): a randomised controlled stage 2b trial. Lancet Reg Health Eur. 2024;39:100864.
  - This is the first randomised controlled trial comparing short-term outcomes of robotic versus open partial pancreaticoduodenectomy.
- Korrel M, Jones LR, van Hilst J, Balzano G, Björnsson B, Boggi U, et al. Minimally invasive versus open distal pancreatectomy for resectable pancreatic cancer (DIPLOMA): an international randomised non-inferiority trial. Lancet Reg Health Eur. 2023;31:100673.
  - This is the first randomised controlled trial showing that minimally invasive distal pancreatectomy is not inferior to open resection.
- Zheng J, Pulvirenti A, Javed AA, Michelakos T, Paniccia A, Lee KK, et al. Minimally Invasive vs. Open Pancreatectomy for Pancreatic Neuroendocrine Tumors: Multi-Institutional 10-Year Experience of 1,023 Patients. J Am Coll Surg. 2022;235(2):315 − 30.
  - This is the largest mulicentre study retropectively comparing perioperative and long-term outcomes after minimally invasive and open resection of pNETs.
- Nießen A, Klaiber U, Lewosinska M, Nickel F, Billmann F, Hinz U, et al. Portal vein resection in pancreatic neuroendocrine neoplasms. Surgery. 2024;175(4):1154-61.
  - This is the largest retrospective single-centre analysis reporting on short- and long-term results after portal vein resection for locally advanced pNETs.
- Gudmundsdottir H, Tomlinson JL, Graham RP, Thiels CA, Warner SG, Smoot RL, et al. Outcomes of pancreatectomy with portomesenteric venous resection and reconstruction for locally advanced pancreatic neuroendocrine neoplasms. HPB (Oxford). 2022;24 (7):1186-93.
  - This is one of the first retrospective analyses reporting on the outcome after portal vein resection in locally advanced pNETs.
- Kaslow SR, Hani L, Cohen SM, Wolfgang CL, Sacks GD, Berman RS, et al. Outcomes after primary tumor resection of metastatic pancreatic neuroendocrine tumors: An analysis of the National Cancer Database. Journal of Surgical Oncology. 2023;128 (2):262 − 70.
  - This is the largest retrospective cohort study evaluating the benefit of primary tumor resection in patients with metastatic pNET.
- Bartsch DK, Windel S, Kanngießer V, Jesinghaus M, Holzer K, Rinke A, et al. Vessel-Sparing Lymphadenectomy Should Be Performed in Small Intestine Neuroendocrine Neoplasms. Cancers (Basel). 2022;14(15).
  - This study retrospectively compared retrograde vessel-sparing lymphadenectomy with conventional resection of siNETs. A comparable amout of lymph nodes harvested, but less small bowel resected was observed when applying this novel technique.
- Li MX, Lopez-Aguiar AG, Poultsides G, Rocha F, Weber S, Fields R, et al. Surgical Treatment of Neuroendocrine Tumors of the Terminal Ileum or Cecum: Ileocecectomy Versus Right Hemicolectomy. J Gastrointest Surg. 2022;26(6):1266-74.
  - This study compared the long-term oncologic outcomes after right-sided hemicolectomy and ileocecectomy for siNETs of the terminal ileum and found similar survival rates despite a lower lymph node harvest after ileocecectomy.
- Yogo A, Paciorek A, Kasai Y, Moon F, Hirose K, Corvera CU, et al. Long-Term Survival Outcomes After Minimally Invasive Surgery for Ileal Neuroendocrine Tumors. Ann Surg Oncol. 2024;31 (9):5507-14.
  - This study evaluated the overall survival after minimally invasive vs. open resection of ileal NETs and found similar long-term outcomes.
- Nesti C, Bräutigam K, Benavent M, Bernal L, Boharoon H, Botling J, et al. Hemicolectomy versus appendectomy for patients with appendiceal neuroendocrine tumours 1–2 cm in size: a retrospective, Europe-wide, pooled cohort study. Lancet Oncol. 2023;24 (2):187 − 94.
  - This is the largest study comparing right-sided hemicolectomy with appendectomy for appendix NETs of intermeditate sizes (1–2 cm).

## Data Availability

No datasets were generated or analysed during the current study.
